# Treatment of nausea in pregnancy: a cross-sectional multinational web-based study of pregnant women and new mothers

**DOI:** 10.1186/s12884-015-0746-2

**Published:** 2015-12-01

**Authors:** Kristine Heitmann, Lone Holst, Angela Lupattelli, Caroline Maltepe, Hedvig Nordeng

**Affiliations:** Department of Global Public Health and Primary Care and Centre for Pharmacy, University of Bergen, Bergen, Norway; Pharmacoepidemiology and Drug Safety Research Group, Department of Pharmacy, School of Pharmacy, University of Oslo, Oslo, Norway; The Motherisk Program, Division of Clinical Pharmacology and Toxicology, The Hospital for Sick Children, Toronto, Canada; Division of Mental Health, Norwegian Institute of Public Health, Oslo, Norway

**Keywords:** Nausea, Pregnancy, Pharmacotherapy, Herbal medicine, Multinational, Internet

## Abstract

**Background:**

The factors related to the treatment of nausea during pregnancy have not yet been investigated in several countries simultaneously. The present study aimed to describe differences in self-reported nausea during pregnancy and the patterns of use for both conventional and herbal medicines across countries. The factors related to nausea and its treatment and the relationships between different self-reported co-morbidities and nausea were also investigated.

**Methods:**

This cross-sectional study used data collected by a web-based questionnaire distributed between October 2011 and February 2012 in several countries within five regions: Western, Northern, and Eastern Europe, North America, and Australia. Women who were pregnant or had a child less than one year old were eligible to participate.

**Results:**

A total of 9113 women were included in the study, whereof 6701 (73.5 %) had experienced nausea during pregnancy. Among respondents with nausea, conventional medicines were used by 1201 (17.9 %) women and herbal medicines by 556 (8.3 %) women. The extent of self-reported nausea and its treatment varied by country. Education, working status, and folic acid use were significantly associated with the use of conventional medicines against nausea. Respondents who had nausea also had a high burden of co-morbidity.

**Conclusion:**

The prevalence of nausea was high across all participating countries but its treatment varied, possibly due to cultural differences and differences in attitudes towards medicines. A high degree of co-morbidity was found among respondents with nausea.

**Electronic supplementary material:**

The online version of this article (doi:10.1186/s12884-015-0746-2) contains supplementary material, which is available to authorized users.

## Background

Nausea and vomiting during pregnancy (NVP) is one of the most common pregnancy-related complaints, affecting millions of pregnant women worldwide each year. Approximately 7 out of 10 women experience nausea during pregnancy [1] and 50 % experience both nausea and vomiting [2–4]. For most patients, symptoms appear around the sixth week of pregnancy and gradually decline during the second trimester, peaking at 8–13 weeks [2, 3]. However, 10 % of women will still experience symptoms after 20–22 weeks of pregnancy [3, 4]. The most severe form of NVP, hyperemesis gravidarum, is characterised by severe and persistent nausea and vomiting leading to weight loss, ketonuria, nutritional deficiencies, dehydration and electrolyte imbalance, often so severe as merits hospitalisation, and affects about 1.1% of the pregnant women [1].

NVP has been shown to greatly impact a woman’s life, negatively affecting daily activities, relationship with partner, parenting, occupation and social functioning [5–7]. Feelings of isolation, fatigue, depression, and helplessness due to nausea have also been described [7–9]. Nausea has been reported to be responsible for 33 % of all sick leave during pregnancy [10]. In the USA and Canada, NVP is a significant economic burden to women and society [11, 12].

Because NVP is often most intense during the first trimester when organogenesis occurs, teratogenic effects are a concern in treatment. This may lead to caution in prescribing and taking conventional medicines to treat this condition, despite the proven safety of use during pregnancy of many medicines. Pregnant women often overestimate the teratogenic risk associated with the use of medicines in general [13]. Consequently, many women may turn to complementary and alternative medicine (CAM) to alleviate their symptoms or choose to not treat their symptoms due to the fear that taking anything during pregnancy may harm the baby. Despite the high prevalence of NVP, little is known about differences in NVP treatments, i.e. conventional and herbal medicines, across countries. An informal survey in various European countries in 1998 found wide variations in the types of treatment used against mild and moderate nausea and vomiting, whereas hyperemesis gravidarum was treated in a similar fashion in the vast majority of countries [14].

No study has investigated the factors related to NVP treatment in several countries simultaneously. Studies are available from various countries, but data collection methods vary [5, 7, 15–20], making direct comparisons impossible. In addition different therapies have been included in the definition of CAM [15, 16, 18, 19].

New possibilities for uniform data collection in several countries are emerging that are advantageous for the field of e-epidemiology [21, 22]. Large potential gains in wellbeing for the mother and society as a whole can be achieved with better knowledge on how nausea in pregnancy is being treated. This study is the first to investigate the factors related to the treatment of nausea during pregnancy at a multinational level.

The present study aimed to describe differences in self-reported nausea during pregnancy, as well as the patterns of use of both conventional and herbal medicine across countries in Western, Northern, and Eastern Europe, North America, and Australia. The study also aimed to investigate the factors related to nausea and its treatment, as well as the relationships between different self-reported co-morbidities and nausea.

## Methods

This cross-sectional study was based on data from a web-based questionnaire covering nausea, medicines against nausea, herbal medicines against nausea, sociodemographic factors, maternal health, and lifestyle during pregnancy [23]. The online questionnaire was distributed simultaneously in 18 countries: Austria, Australia, Croatia, Canada, France, Finland, Iceland, Italy, Norway, Poland, Russia, Serbia, Slovenia, Sweden, Switzerland, the Netherlands, United Kingdom, and USA. The original data file consisted of 9459 women, including 346 South American women who accessed the questionnaire via North American web-sites [23]. For this sub-study, the 346 women from South America were excluded in effort to reduce selection bias as this was considered a special group of women, resulting in a final study population of 9113 women.

Women who were pregnant or had a child who was less than one year old were eligible to participate in the study. An advert containing a link to the online self-completed questionnaire was posted on commonly visited pregnancy and baby related websites in the participating countries. National coordinators selected the most relevant national websites, social networks, and pregnancy forums [23]. The questionnaire was available for 2 months in each participating country between 1st of October 2011 and 29th of February 2012.

The questionnaire was originally developed in Norwegian and English before being translated into the relevant languages, and is available online as an appendix to the paper by Lupattelli et al. [23]. A pilot study was performed during September 2011 in Norway, Finland, Italy, and Sweden (*n* = 47) but resulted in no major changes to the questionnaire. All national coordinators assured the quality of their version of the questionnaire.

The representativeness of the study population was assessed by comparing the sociodemographic and lifestyle characteristics (i.e., age, marital status, education, and smoking) of the study population to the general birthing population in the corresponding country. The similarities were satisfactory with the exception that the study participants were generally more educated than the general birthing population, as described in detail elsewhere [23].

### Measures of nausea, health disorders, and conventional and herbal medicines use during pregnancy

The respondents were presented with a list of questions related to different health disorders/short-term illnesses during pregnancy, including nausea, and asked if they had any of these illnesses. In case of an affirmative response, the respondents were asked about medicine use related to each individual illness. The medicines used were reported in free-text entry fields. The timing of use for both conventional and herbal medicines could also be reported and were defined by the three possible exposure windows included in the questionnaire: weeks 1–12 (first trimester); weeks 13–24 (second trimester), and week 25 to delivery (third trimester). A list of chronic disorders was also presented to the respondents, including cardiovascular and rheumatic disorders, diabetes and epilepsy, and an open-ended option. Furthermore, the women were presented with a question on sick leave during pregnancy (dichotomised yes/no).

In addition to the standardised questions about medicine use for specific illnesses, the respondents were questioned about over-the-counter (OTC) medicine use during pregnancy, including OTC medicines against nausea, and the timing of use. A medicine was defined as a single product containing one or more active ingredients. The main active ingredient(s) and formulation of the branded medicinal product were identified for each specific trademark name and recorded using either the national medicine database or the Martindale textbook [24]. All medicines were then coded into the corresponding Anatomical Therapeutic Chemical (ATC) codes in accordance with the World Health Organization (WHO) ATC index [25]. Whenever possible, the 5th level of the ATC was used.

Any use of herbal medicines was specifically requested, including the name of the product, reason for its use, and the timing of use during pregnancy. The name of the herbal medicine and the reason for its use were reported as free-text entry fields. Herbal medicine could also be reported under the disease-specific questions and the questions about OTC medicine. Herbal medicines were identified by name and coded in accordance with a pre-determined list of herbs [26].

The respondents were classified as having nausea during pregnancy if they reported having had nausea when questioned about short-term illnesses, if they reported any use of OTC medicines against nausea, or if they gave nausea as an indication for the use of herbal or homeopathic medicines.

### Sociodemographic and lifestyle variables

The following variables were explored in relation to nausea and the use of conventional medicines: region of residence, maternal age, parity, marital status, education, working status, smoking during pregnancy, use of folic acid, and multiple pregnancy. Sociodemographic variables were categorised as presented in Table 1.

**Table 1:** Factors related to nausea and treatment of nausea

	Total population	Total Nausea	Crude OR (95 % CI)	Adjusted OR (95 % CI)^b^	Nausea, conventional medicines against nausea	Crude OR (95 % CI)	Adjusted OR (95 % CI)^b^
						Nausea, conventional medicines against nausea vs. Nausea, no treatment^c^	Nausea, conventional medicines against nausea vs. Nausea, no treatment^c^
	*n* = 9113 (% of 9113)	*n* = 6701 (% of total in row)	Nausea vs. no nausea	Nausea vs. no nausea	*n* = 1201 (% of total nausea in row)		
Age, years							
≤24	1413 (15.5)	1053 (74.5)	1.1 (1.0-1.2)	1.2 (1.0-1.3)	183 (17.4)	0.9 (0.7-1.2)	0.9 (0.6-1.2)
25-29	3061 (33.6)	2236 (73.0)	1	1	417 (18.6)	1	1
30-34	2939 (32.3)	2191 (74.5)	1.1 (1.0-1.2)	1.0 (0.9-1.2)	399 (18.2)	1.0 (0.8-1.2)	1.0 (0.7-1.3)
≥35	1630 (17.9)	1162 (71.3)	0.9 (0.7-1.2)	0.8 (0.6-1.1)	185 (15.9)	0.8 (0.7-1.0)	0.9 (0.7-1.0)
Marital status							
Married/cohabiting	8578 (94.1)	6322 (73.7)	1	1	1137 (18.0)	1	1
Single/divorced/other	535 (5.9)	379 (70.8)	**0.9 (0.8-0.9)**	0.9 (0.9-1.0)	64 (16.9)	0.9 (0.8-1.1)	0.9 (0.6-1.2)
Parity							
0 previous live births	4602 (50.5)	3211 (69.8)	1	1	571 (17.8)	1	1
≥1 previous live births	4511 (49.5)	3490 (77.4)	**1.5 (1.4-1.6)**	**1.5 (1.3-1.7)**	630 (18.1)	1.0 (0.7-1.4)	1.0 (0.7-1.4)
Education							
Primary school	380 (4.2)	288 (75.8)	1.2 (0.9-1.6)	1.2 (0.8-1.8)	74 (25.7)	**1.6 (1.2-2.1)**	**1.5 (1.1-2.3)**
High school	2574 (28.2)	1901 (73.9)	1.0 (0.9-1.3)	1.1 (0.8-1.4)	309 (16.3)	0.9 (0.8-1.0)	0.9 (0.8-1.1)
University or college	5120 (56.2)	3738 (73.0)	1	1	657 (17.6)	1	1
Other education	1039 (11.4)	774 (74.5)	1.1 (1.0-1.2)	1.1 (0.9-1.3)	161 (20.8)	1.2 (1.0-1.6)	1.2 (1.0-1.6)
Working status							
Employed, but not as health care personnel	5417 (59.4)	3874 (71.5)	1	1	629 (16.2)	1	1
Health care personnel	1236 (13.6)	961 (77.8)	**1.4 (1.2-1.7)**	**1.3 (1.1-1.6)**	216 (22.5)	**1.4 (1.2-1.7)**	**1.4 (1.2-1.6)**
Unemployed	1991 (21.8)	1536 (77.1)	**1.3 (1.2-1.5)**	**1.3 (1.2-1.4)**	292 (19.0)	1.2 (1.0-1.3)	1.2 (1.0-1.5)
Other	457 (5.0)	323 (70.7)	1.0 (0.9-1.0)	0.9 (0.9-1.0)	62 (19.2)	1.2 (1.0-1.5)	1.1 (0.9-1.3)
Use of folic acid							
Before the pregnancy	311 (3.4)	237 (76.2)	1.0 (0.9-1.2)	1.0 (0.9-1.2)	58 (24.5)	**1.5 (1.3-1.9)**	**1.6 (1.3-1.9)**
Before and during pregnancy	4077 (44.7)	3072 (75.3)	1	1	552 (18.0)	1	1
Only during pregnancy	3929 (43.1)	2821 (71.8)	0.8 (0.7-1.0)	**0.8 (0.7-1.0)**	488 (17.3)	0.9 (0.8-1.1)	0.9 (0.8-1.1)
No	716 (7.9)	517 (72.2)	0.9 (0.7-1.1)	0.8 (0.7-1.1)	93 (18.0)	1.0 (0.6-1.7)	1.0 (0.6-1.7)
Smoking during pregnancy							
No	8227 (90.3)	6125 (74.4)	1	1	1092 (17.8)	1	1
Yes	864 (9.5)	560 (64.8)	**0.6 (0.6-0.7)**	**0.6 (0.5-0.7)**	105 (18.8)	1.0 (0.8-1.3)	1.0 (0.8-1.3)
Pregnant population	*n* = 4938 (% of 4938)	*n* = 3762 (% of total in row)		**Adjusted OR (95 % CI)** ^**d**^	*n* = 657 (% of total nausea in row)		**Adjusted OR (95 % CI)** ^**d**^
Multiple pregnancy^a^							
No	4817 (97.5)	3667 (76.1)	1	1	636 (17.3)	1	1
Yes	76 (1.5)	65 (85.5)	1.9 (0.8-4.5)	2.0 (0.8-4.9)	17 (26.2)	**1.6 (1.1-2.3)**	**1.6 (1.1-2.4)**

Numbers do not add up due to missing numbers

Significant findings are in bold

*Abbreviations*: *OR* odds ratio; *CI* confidence interval

^a^This question was only posed to pregnant women (*n* = 4938). Only pregnant women are included in the analysis

^b^Adjusted for all other variables in the table with the exception of “multiple pregnancy”

^c^Nausea, no treatment includes women with nausea not using any of the following treatments against nausea: conventional medicines, herbal medicines, homeopathic medicines and dietary supplements

^d^Only pregnant women are included in the analysis. Variables included in the model: age, marital status, parity, education, working status, use of folic acid, and smoking

### Measurements of maternal mental health

Symptoms of depression were measured by the Edinburgh Postnatal Depression Scale (EPDS), a self-rating 10-item scale initially developed by Cox et al. to detect postnatal depression [27]. However, the scale has also been validated as a screening tool for major depression in pregnant women with satisfactory results and has been used in several studies in various countries [28]. Cut-off scores of 11, 10 and 10 applied at weeks 12, 24, and 36 of pregnancy, respectively, resulted in 79 %, 70 %, and 76 % sensitivity, respectively, and 97 %, 96 %, and 94 % specificity, respectively [28]. Each question has four different options scored as 0, 1, 2, or 3. The scale rates the intensity of depressive symptoms over the previous 7 days. The total score ranges between 0 and 30. Having symptoms of depression was defined as having a total EPDS score ≥13 [27]. Validated translated versions of the original EPDS were available for eight languages other than English: Dutch, French, German, Icelandic, Norwegian, Slovenian, Spanish, and Swedish [29]. The Serbian version was developed by two independent linguistic experts, who carried out translations and back-translations. Any discrepancies between the back-translated and original EPDS were identified and corrected. For the remaining five languages, the translated versions used in previous studies were utilised [30–33].

### Statistical analysis

Descriptive statistics were used to calculate the prevalence of conventional and herbal medicines use against nausea during pregnancy and presented as percentages. Univariate and multivariate generalised estimating equation (GEE) analyses were performed to explore potential significant associations between the maternal characteristics listed in Table 1 and the use of conventional medicines against nausea. The GEE with the binary logistic model was used to correct for clustering on region of residency. Odds ratios (ORs) are presented with 95 % confidence intervals (CIs). All variables in Table 1, with the exception of multiple pregnancy, were included in the multivariate models.

**Table 2:** Most common treatments against nausea by region and country

Region or country	Nausea	Use of any treatment^a^	Use of conventional medicines	Most frequently used conventional medicine	Use of herbal medicines	Most frequently used herbal medicine
	n (%)	n (%)	n (%)	(n)	n (%)	(n)
Western Europe (*n* = 3201)	2338 (73.0)	736 (23.0)	449 (14.0)	Antihistamines (174)	230 (7.2)	Ginger (203)
Austria (*n* = 82)	54 (65.9)	15 (18.3)	7 (8.5)	Antihistamines (3) and metoclopramide (3)	8 (9.8)	Ginger (8)
France (*n* = 374)	263 (70.3)	140 (37.4)	101 (27.0)	Metoclopramide (43)	7 (1.9)	Ginger (3)
Italy (*n* = 926)	645 (69.7)	193 (20.8)	77 (8.3)	Metoclopramide (25)	80 (8.6)	Ginger (71)
The Netherlands (*n* = 81)	58 (71.6)	14 (17.3)	12 (14.8)	Antihistamines (8)	3 (3.7)	Ginger (3)
Switzerland (*n* = 618)	436 (70.6)	213 (34.5)	165 (26.7)	Antihistamines (118)	49 (7.9)	Ginger (43)
United Kingdom (*n* = 1120)	882 (78.8)	161 (14.4)	87 (7.8)	Antihistamines (35)	83 (7.4)	Ginger (75)
Northern Europe (*n* = 2820)	2259 (80.1)	533 (18.9)	417 (14.8)	Antihistamines (316)	112 (4.0)	Ginger (107)
Finland (*n* = 574)	453 (78.9)	47 (8.2)	37 (6.4)	Metoclopramide (17)	3 (0.5)	Ginger (3)
Iceland (*n* = 71)	60 (84.5)	26 (36.6)	17 (23.9)	Antihistamines (12)	12 (16.9)	Ginger (11)
Norway (*n* = 1288)	1028 (79.8)	199 (15.5)	120 (9.3)	Antihistamines (74)	95 (7.4)	Ginger (92)
Sweden (*n* = 887)	718 (80.9)	261 (29.4)	243 (27.4)	Antihistamines (219)	2 (0.2)	Ginger (1) and black pepper (1)
Eastern Europe (*n* = 2342)	1512 (64.6)	303 (12.9)	146 (6.2)	Antacids (56)	121 (5.2)	Ginger (69)
Croatia (*n* = 286)	182 (63.6)	27 (9.4)	14 (4.9)	Antacids (5)	1 (0.3)	Other herbal products (1)
Poland (*n* = 679)	447 (65.8)	81 (11.9)	37 (5.4)	Antihistamines (16)	43 (6.3)	Ginger (36)
Russia (*n* = 1008)	625 (62.0)	146 (14.5)	81 (8.0)	Antacids (29)	59 (5.9)	Artichoke (28)
Serbia (*n* = 220)	144 (65.5)	29 (13.2)	13 (5.9)	Antacids (7)	0	-
Slovenia (*n* = 149)	114 (76.5)	20 (13.4)	1 (0.7)	Antacids (1)	18 (12.1)	Ginger (16)
North-America (*n* = 533)	415 (77.9)	171 (32.1)	137 (25.7)	Antihistamines (96)	46 (8.6)	Ginger (41)
Canada (*n* = 236)	177 (75.0)	85 (36.0)	74 (31.4)	Antihistamines (68)	19 (8.1)	Ginger (18)
USA (*n* = 297)	238 (80.1)	86 (29.0)	63 (21.2)	Ondansetron (29)	27 (9.1)	Ginger (23)
Australia (*n* = 217)	177 (81.6)	85 (39.2)	52 (24.0)	Metoclopramide (32)	47 (21.7)	Ginger (46)
Total population (*n* = 9113)	6701 (73.5)	1828 (20.1)	1201 (13.2)	Antihistamines (613)	556 (6.1)	Ginger (466)

^a^Including conventional medicines, herbal medicines, homeopathic medicines and dietary supplements

Antacids are defined as all medicines with ATC-code A02

Antihistamines are defined as all medicines with ATC-code R06

Univariate and multivariate GEE analyses were also used to explore the relationships between co-morbidity and nausea and its treatment. First, univariate analyses were performed. Then full multivariate models were built including all variables presented in Table 1. Reduced models were fit by excluding non-significant variables (significance level: p < 0.05), unless removal of the variable caused a >10 % change in the effect estimates. Sub analyses including the EPDS were restricted to pregnant women only, as the EPDS is based on symptoms during the prior week. Moreover, stratified analyses on timing of gestation (during first trimester versus after first trimester) were performed when studying the association between nausea and comorbidity.

All statistical analyses were performed using Statistical Package for the Social Sciences (SPSS) version 20.0 (IBM SPSS Statistics 20) for Windows (SPSS, Chicago, IL, USA).

### Ethics

Before entering the online questionnaire, the respondents had to 1) read the study description in which the study objectives, the participants’ right to withdraw at any time, and contact persons in the applicable country were presented, and 2) answer the following question: “Are you willing to participate in the study?” If the woman ticked “yes” as the answer it was considered informed consent.

The study was approved by the Regional Ethics Committee, Region South-East in Norway, and the relevant Ethics Boards in each specific country when required [23]. Complementary ethical approval was required and obtained from the Faculty of Medicine and Health Science Research Ethics Committee of the University of East Anglia in the UK, The National Bioethics Committee in Iceland and The Scientific Ethic Board, Provincial Health Service of Trento in Italy.The STROBE statements were used when writing this paper (Additional file 1).

## Results

During the 2-month study period in each country, a total of 9113 women were included in the study. Respondents who were residents of Europe (Western, *n* = 3201; Northern, *n* = 2820; Eastern, *n* = 2342) constituted the largest proportion of the total study population, followed by North America (*n* = 533) and Australia (*n* = 217).

At the time of completing the questionnaire, 4938 (54.2 %) of the women were pregnant and 4175 (45.8 %) had given birth during the previous year. Among the pregnant respondents, 1067 (21.6 %), 1656 (33.5 %), and 2214 (44.8 %) were in the first, second, and third trimester of their pregnancy, respectively, and 182 (3.7 %) were less than 6 weeks pregnant. A total of 1913 (45.8 %) of the mothers had an infant less than 24 weeks of age.

Among the respondents, 6701 (73.5 %) had experienced nausea during pregnancy; 1828 (27.3 %) used some form of treatment against nausea and 4873 (72.7 %) did not. Conventional medicines against nausea were used by 1201 (17.9 %) of the women and herbal medicines by 556 (8.3 %).

**Fig. 1:**
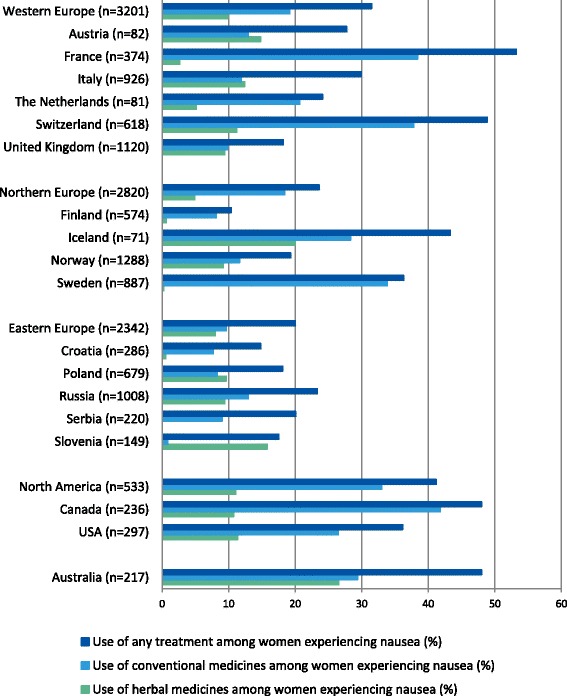
Use of treatment against nausea among women experiencing nausea

Both the prevalence of nausea and its treatment varied by country and region (Table 2 and Fig. 1). The prevalence of nausea ranged from 62.0 % in Russia to 84.5 % in Iceland. The proportion of respondents treated among those who suffered from nausea ranged from 10.4 % in Finland to 53.2 % in France; the next highest proportions were in Switzerland (48.9 %), Canada (48.0 %), and Australia (48.0 %). In 11 countries the treatment rates were below 30 % (Fig. 1). Among the regions, Australia (48.0 %) and North America (41.2 %) had the highest rates of treatment.

The most commonly used conventional medicines against nausea in the total population were antihistamines, which were used by 613 respondents (6.7 %) (Table 2 and Additional file 2). Metoclopramide was the second most commonly used medicine with 268 respondents (2.9 %). Antacids (ATC-group A02), ondansetron, and domperidone were used by 176 (2.6 %), 54 (0.6 %), and 48 (0.5 %) respondents, respectively. Conventional medicines were most commonly used against nausea in Canada, France, Switzerland and Sweden. The type of conventional medicine most commonly used among women with nausea differed by region and country, but in the majority of countries it was either antihistamines or metoclopramide. An exception was ondansetron, which was the most commonly used medicine in the United States, closely followed by antihistamines. In Croatia, Russia, Serbia, and Slovenia the most common medicines were antacids, despite heartburn and reflux problems being less prevalent in these countries (60.5 %, 59.6 %, 52.7 %, and 57.7 %, respectively) compared to the total population (66.0 %). The countries in Eastern Europe had a low frequency of conventional medicines use. One respondent from Slovenia reported the use of conventional medicines. Interestingly, metopimazine was reported to be used against nausea by 29 respondents, all in France. In Canada, 54 respondents had used Diclectin®, which is a combination of the antihistamine doxylamine and pyridoxine.

Among the five regions, Australia had the highest frequency of herbal medicine use (21.7 %). Ginger was the most commonly used herbal medicine in the total population (5.1 %) and in most regions and countries. However, in Russia the most commonly used herbal medicine was artichoke (2.8 %). In most countries, herbal medicines were used to a lesser extent than conventional medicines, with the exception of Slovenia, Poland, Austria, and Italy.

Maternal characteristics as predictors of nausea and the use of conventional medicines against nausea during pregnancy are shown in Table 1. Respondents who had more than one previous live birth, worked as health care personnel, or were unemployed were more likely to experience nausea, whereas respondents who used folic acid during pregnancy only or smoked during pregnancy were less likely to experience nausea according to adjusted models. Respondents who had primary school as their highest completed education, were health care personnel, or had used folic acid before the pregnancy were more likely to have used conventional medicines against nausea than respondents with characteristics within the respective reference categories. Multiple pregnancy was also associated with use of medicines against nausea.

Women who experienced nausea during pregnancy were more likely to have any of the acute short-term illnesses listed in Table 3. These respondents were also more likely to have four or more co-morbidities in terms of acute short-term illnesses, any chronic illness, or to have taken sick leave during pregnancy. This pattern was similar when comparing respondents who had nausea and used conventional medicines against nausea to the respondents who experienced nausea without using any treatment. However, the effect estimates were generally lower than for nausea alone. Sub analyses including only respondents pregnant at the time of participation in the study revealed that respondents who experienced nausea were more likely to have symptoms of depression (EPDS score ≥13) than respondents without nausea. This was also true among respondents who had nausea and used conventional medicines compared to respondents who experienced nausea without treatment.

**Table 3:** Co-morbidities according to nausea in pregnancy and its treatment

	Total population	Nausea	Crude OR (95 % CI)	Adjusted OR (95 % CI)	Nausea, conventional medicines against nausea	Crude OR (95 % CI)	Adjusted OR (95 % CI)
	n = 9113 (% of 9113)	n = 6701 (% of 6701)	Nausea vs. No nausea	Nausea vs. No nausea	n = 1201 (% of 1201)	Nausea, conventional medicines against nausea vs. Nausea, no treatment^a^	Nausea, conventional medicines against nausea vs. Nausea, no treatment^a^
Heartburn or reflux problems	6011 (66.0)	4703 (70.2)	**2.0 (1.8-2.1)**	**2.0 (1.8-2.1)** ^**e**^	908 (75.6)	**1.4 (1.2-1.7)**	**1.4 (1.1-1.7)** ^f^
Sleeping problems	5207 (57.1)	4107 (61.3)	**1.9 (1.7-2.1)**	**1.9 (1.8-2.1)** ^**e**^	798 (66.4)	**1.3 (1.2-1.5)**	**1.3 (1.2-1.5)** ^f^
Constipation	4757 (52.2)	3686 (55.0)	**1.5 (1.4-1.7)**	**1.5 (1.3-1.7)** ^e^	678 (56.5)	1.1 (0.9-1.3)	1.1 (0.9-1.3)^f^
Headache	5014 (55.0)	3983 (59.4)	**2.0 (1.9-2.0)**	**1.9 (1.8-2.0)** ^e^	767 (63.9)	**1.3 (1.2-1.3)**	**1.3 (1.2-1.4)** ^f^
Pain in neck, back or pelvic girdle	6227 (68.3)	4798 (71.6)	**1.7 (1.5-2.0)**	**1.7 (1.5-1.9)** ^e^	910 (75.8)	**1.3 (1.1-1.5)**	**1.3 (1.1-1.6)** ^f^
Any chronic illness^b^	2273 (24.9)	1738 (25.9)	**1.2 (1.2-1.3)**	**1.2 (1.2-1.3)** ^e^	361 (30.1)	**1.2 (1.2-1.3)**	**1.3 (1.0-1.6)** ^f^
≥4 co-morbidities^c^	5257 (57.7)	4245 (63.3)	**2.4 (2.2-2.6)**	**2.3 (2.2-2.5)** ^e^	849 (70.7)	**1.5 (1.3-1.8)**	**1.5 (1.3-1.8)** ^f^
Sick leave during pregnancy	3956 (43.4)	3001 (44.8)	**1.2 (1.0-1.5)**	**1.3 (1.1-1.6)** ^e^	625 (52.0)	**1.4 (1.1-2.0)**	**1.5 (1.1-2.2)** ^f^
Pregnant population	n = 4938 (% of 4938)	n = 3762 (% of 3762)	Crude OR (95 % CI)	Adjusted OR (95 % CI)	n = 657 (% of 657)	Crude OR (95 % CI)	Adjusted OR (95 % CI)
Symptoms of depression during pregnancy^d^	863 (17.5)	718 (19.1)	**1.7 (1.6-1-8)**	**1.5 (1.4-1.6)** ^**g**^	183 (27.9)	**1.9 (1.5-2.3)**	**1.9 (1.5-2.4)** ^**h**^

*Abbreviations*: *OR* odds ratio; *CI* confidence interval

Significant findings are in bold

^a^Nausea, no treatment: Includes women with nausea not using any of the following treatments against nausea: conventional medicines, herbal medicines, homeopathic medicines and dietary supplements.

^b^Any chronic illness includes asthma, allergy, hypothyroidism, rheumatic disorders, diabetes, epilepsy, depression, anxiety, cardiovascular diseases, and other

^c^≥ 4 co-morbidities includes women who reported experiencing more than three of the following disorders during pregnancy: heartburn or reflux problems, constipation, common cold, urinary tract infections, other infections, pain in the neck, back, or pelvic girdle, headache, and sleeping problems.

^d^Only women who were pregnant at the time of participating are included (*n* = 4938). Symptoms of depression were measured by the Edinburgh Postnatal Depression Scale. Symptoms of depression were defined as an EPDS score of ≥ 13

^e^Adjusted for age, parity, working status, use of folic acid, and smoking during pregnancy

^f^Adjusted for age, education, working status, and use of folic acid

^g^Adjusted for age, parity, working status, smoking during pregnancy, and ≥4 co-morbidities

^h^Adjusted for education, working status, use of folic acid, multiple pregnancy, and ≥4 co-morbidities

In an additional sub analysis we found that time of gestation acted as a plausible effect modifier of the association between medicated nausea and comorbidity. Specifically, respondents early in their pregnancy (≤1 trimester) who treated nausea with conventional medicines presented a significant 3.1-, 2.8-, and 2.1-fold increased likelihood of taking sick leave (crude OR: 3.1, 95 % CI: 1.8-5.5), having depressive symptoms during pregnancy (crude OR: 2.8, 95 % CI: 1.7-4.6), and having heartburn and reflux problems (crude OR: 2.1, 95 % CI: 1.6-2.6), respectively, compared to respondents with non-medicated nausea. Such measures of association were of a much smaller magnitude (30-70 % increased likelihood) in the stratum comprising only respondents later in their pregnancy (>1 trimester). Similar results were observed when respondents with nausea were compared to those without nausea (data not shown).

## Discussion

Variations were found across countries and regions in the prevalence of nausea, treatment rates, and types of treatment used against nausea during pregnancy. Cultural differences reflected in different treatment traditions, differences between countries with respect to the women’s and general practitioners’ willingness to treat, and variations in access to prenatal care and treatments and their relative costs may explain several of our findings. Among respondents suffering from nausea, less than one in three used any form of treatment, and only 18 % had used any medicine against this complaint. We do not have data on the severity of nausea, and the respondents may generally suffer from mild symptoms that are sufficiently managed by non-pharmacological treatments, such as dietary changes. However, the low prevalence of treatment may also be explained by a reluctance of many general practitioners to treat these women [34], or by an overestimation of the risk of medicines among pregnant women with nausea [35]. The overall prevalence of nausea (73.5 %) in this study is in accordance with a recent meta-analysis of NVP including 59 studies from various countries [1].

Canada, followed by France, Switzerland and Sweden, had the highest prevalence of conventional medicines use against nausea. This finding may be due to the clear and well known guidelines in this country [36, 37] and the antiemetic Diclectin®, which is approved for use against NVP. Therefore, simplifying the identification of safe and effective treatments may possibly increase the use of treatment. Among the European countries, medicine use was highest in France, which is in line with the results of a study of drug utilisation in pregnancy that included six European Registries of Congenital Anomalies in four European countries: France, Great Britain, Italy, and the Netherlands [38]. The authors of the study found that the two centres in France had the highest prevalence of medicine use (80.8 % and 74.2 %), and that antinauseants were the most frequently consumed drugs in this country (20.9 % and 15.0 %) [38]. We found that several French respondents used metopimazine, a dopamine antagonist, which was not reported in any of the other countries. This finding is in accordance with a comparative study by Einarson and colleagues in 1998 in which France was the only country to list metopimazine as a treatment option [14]. In Sweden, antihistamines were the most frequently used medicines against nausea, which is also in line with previous findings [17].

Antihistamines and metoclopramide were the most commonly used conventional medicines against nausea in the vast majority of the countries. The exceptions were the USA, Croatia, Russia, Serbia, and Slovenia, where ondansetron (USA) and antacids (Croatia, Russia, Serbia, and Slovenia) were the most commonly used medicines against nausea. This is in line with findings of a study of hyperemesis gravidarum treatments detecting inter-country variations of frequency of different treatments of which serotonin inhibitors were most frequently used in the USA, antihistamines in Canada, whereas Australia had the highest reported use of promotility agents such as metoclopramide [39]. Meta-analyses and epidemiological studies have not found a higher risk of malformations with antihistamines and metoclopramide [40–43], and antihistamines are regarded as a first line treatment according to guidelines in both North America and Europe [36, 37, 44–46]. Recently, the safety of metoclopramide and ondansetron has been questioned [47, 48]. In July 2013, the European Medicines Agency (EMA) recommended changing the use of metoclopramide to 10 mg three times a day up to 5 days to reduce the risk of extrapyramidal side effects [48]. As this is seldom long enough to treat NVP, the change will limit this medicine’s usefulness for nausea and vomiting in the pregnant population. In 2011, the U.S. Food and Drug Administration (FDA) raised concerns over cardiovascular safety, suggesting that ondansetron could cause prolonged QT interval, which can lead to Torsade de Pointes [49]. Electrocardiogram (ECG) monitoring in patients with electrolyte abnormalities is advised. A recent Danish registry study of ondansetron use during the first trimester did not detect any increased risk of malformations [50]. Another unpublished study based on the same registries detected a 2-fold increase in the prevalence of major congenital heart defects after exposure to ondansetron [51]. Notably, the data in our study were collected during winter 2011–2012. Therefore, the medication utilisation pattern may have changed due to the warnings issued by the EMA (2013) and FDA (2011) with respect to metoclopramide and ondansetron, respectively.

Australia had the highest rate of herbal medicine use against nausea, followed by Iceland and Slovenia. Australia has previously been reported to have a high prevalence of herbal medicine and CAM use in pregnancy in general [52, 53], and also more specifically a high use of ginger during pregnancy [52]. The results with respect to Slovenia were special; 12.1 % of respondents had used herbal medicine, but only one respondent had used a conventional medicine against nausea. This finding may indicate a long tradition of herbal medicine in Slovenia or a lack of access to conventional medicines.

Ginger was the dominant herbal medicine used against nausea. Ginger has been reported to be more effective than placebo and equally effective as vitamin B6 and dimenhydrinate against nausea in pregnancy [54]. With respect to safety, a cohort study with 1020 ginger-exposed pregnancies (466 in the first trimester) found no increased risk of malformations, stillbirth/perinatal death, low birth weight, preterm birth, or low Apgar score [16]. Russia was the only country to report the use of artichoke against nausea. However, no studies of artichoke use in pregnancy were found, though artichoke has been observed to have an antiemetic effect in outpatients with dyspeptic syndrome [55].

Various maternal characteristics were associated with nausea and its treatment. Having more than one previous live birth was associated with nausea, probably because having additional children results in less time to rest and relieve the nausea. This finding is in accordance with previous research [56, 57], but the data are conflicting [4]. Other factors associated with nausea were working as health care personnel or being unemployed, which is in line with previous research that found an association between being a housewife or out of work and nausea [57, 58]. Respondents who smoked during pregnancy or who used folic acid during pregnancy were less likely to report nausea. Decreased risk of nausea among smokers was observed in several earlier studies [4, 56, 57]. Use of vitamins in early pregnancy was previously found to be protective against nausea [57, 59]. Women who take folic acid before, as well as during, pregnancy are most likely planning to become pregnant, and this may imply that they are more attentive to early symptoms of pregnancy than women who use folic acid only during pregnancy.

Respondents who had a lower education were more likely to use medicines against nausea. This finding is in accordance with a Swedish study [17]. Respondents working as health care professionals were also more likely to use medicines against nausea, which can be explained by this group being aware of safe and effective treatment options for nausea. Multiple pregnancy was associated with the use of medicines. This may indicate that use of medicines may act as a marker of severe forms of nausea, as it is previously found that multiple pregnancy increase the risk of nausea [56]. In addition, the severity of NVP symptoms has been associated with the use of antiemetics [7, 60].

We found a high burden of co-morbidity among respondents experiencing nausea during pregnancy. The association with symptoms of depression and sick leave in particular warrants attention. Women who suffer from any pregnancy-related complaint may tend to seek information on the internet to a greater extent than women who feel well. Therefore, the respondents may be seeking information and responding at the peak of their discomfort. However, symptoms of depression have also been associated with nausea during pregnancy [7, 9, 60]. Similarly, the association with heartburn and reflux problems is in agreement with previous studies [61]. Clinicians should be aware of the high degree of co-morbidity with nausea and routinely ask women with nausea whether they have reflux problems or other pregnancy-related ailments.

Our findings indicate that women who have nausea in early pregnancy, especially those who treat nausea with medicines, have a high likelihood of experiencing depressive symptoms, heartburn and reflux problems, and taking sick leave. This is an important clinical finding and emphasises how debilitating nausea during pregnancy can be for these women. General practitioners in contact with women with NVP should be aware of the high degree of co-morbidity, examine these women for symptoms of depression and heartburn and reflux problems, and treat these conditions if present. Special attention should be paid to women in early pregnancy. Treating heartburn and reflux problems may alleviate symptoms of NVP and increase the women’s wellbeing [62]. Major guidelines suggest antacids as adjunctive therapy against NVP [36, 37, 44].

This study has several strengths and limitations that should be acknowledged. This is the first multinational study to simultaneously collect data on the prevalence of nausea and its treatment, which enables direct comparisons between countries and regions. A large number of women from a variety of countries in different regions of the world were reached due to the utilisation of a web-based questionnaire posted on various pregnancy-related websites. These data provide valuable insights into the prevalence of nausea and the treatment of this complaint across countries and regions. Furthermore, the study population was reasonably comparable to the general birthing population with respect to age, parity, and smoking habits, though the women in the study population had a higher education on average [23]. However, the possibility that the respondents differ from the general birthing population in ways that our analysis cannot control for cannot be excluded. In some of the countries (Australia, Canada, France, Russia, the Netherlands, and the USA), the study sample was a small proportion of the general birthing population. For these specific countries, our findings should be generalised with caution.

There are some other limitations that need to be addressed. First, a conventional response rate could not be calculated because the questionnaire was only accessible through websites. However, epidemiological studies have indicated that web-based recruitment methods have reasonable validity [63, 64]. In addition, women of childbearing age generally have a relatively high internet penetration rate [65–67]. The fact that we found a prevalence of nausea very similar to the prevalence reported in the literature, and that the comparison with the birthing population in each participating country had high external validity, supports our approach. However, the higher education of the respondents may have had an impact on their choice of treatment. Second, including women at an early stage in their pregnancy may underestimate the prevalence of nausea, as this complaint often does not occur before gestational weeks 6–8. However, this only applies to the 182 women (2.0 %) who were less than 6 weeks pregnant at the time of participation. Thirdly, although we tried to minimise the risk of recall bias by excluding women with a youngest child aged >1 year, this risk cannot be ruled out. In addition, the EPDS was only measured at one time point during the pregnancy and two time points are considered more valid [29]. Finally, we lack information on the severity of nausea and our results should be interpreted with these limitations in mind.

## Conclusions

The prevalence of nausea was high across all participating countries, but its treatment varied, possibly due to cultural differences and differences in attitudes towards medicines. Women who reported nausea also had a high burden of co-morbidity, especially heartburn and reflux symptoms. The association with symptoms of depression and sick leave warrants attention. These findings will be helpful to health care personnel involved in the care of pregnant women with nausea.

## Additional files

Additional file 1:
**STROBE Statement—Checklist of items that should be included in reports of** ***cross-sectional studies.*** (PDF 96 kb) Additional file 2:
**Most common medicines used against nausea on 1st and 2nd ATC level according to timing of use in pregnancy among women with nausea in pregnancy.** Description of data: A table containing the most common medicines on the 1st and 2nd ATC levels used against nausea according to the timing of use in pregnancy among women with nausea. (PDF 21 kb)

## Abbreviations used

NVP: Nausea and vomiting during pregnancy
CAM: Complementary and alternative medicine
OTC medicine: Over-the-counter medicine
ATC: Anatomical Therapeutic Chemical
WHO: World Health Organization
EPDS: Edinburgh Postnatal Depression Scale
GEE analyses: Generalised estimating equation analyses
OR: Odds ratio
CI: Confidence interval
SPSS: Statistical Package for the Social Sciences
EMA: the European Medicines Agency
FDA: the U.S. Food and Drug Administration
ECG: Electrocardiogram

## References

[1] Einarson TR, Piwko C, Koren G (2013). Quantifying the global rates of nausea and vomiting of pregnancy: a meta analysis. J Popul Ther Clin Pharmacol..

[2] Gadsby R, Barnie-Adshead AM, Jagger C (1993). A prospective study of nausea and vomiting during pregnancy. Br J Gen Pract.

[3] Lacroix R, Eason E, Melzack R (2000). Nausea and vomiting during pregnancy: A prospective study of its frequency, intensity, and patterns of change. Am J Obstet Gynecol.

[4] Klebanoff MA, Koslowe PA, Kaslow R, Rhoads GG (1985). Epidemiology of vomiting in early pregnancy. Obstet Gynecol.

[5] Smith C, Crowther C, Beilby J, Dandeaux J (2000). The impact of nausea and vomiting on women: a burden of early pregnancy. Aust N Z J Obstet Gynaecol.

[6] Attard CL, Kohli MA, Coleman S, Bradley C, Hux M, Atanackovic G (2002). The burden of illness of severe nausea and vomiting of pregnancy in the United States. Am J Obstet Gynecol.

[7] Mazzotta P, Stewart D, Atanackovic G, Koren G, Magee LA (2000). Psychosocial morbidity among women with nausea and vomiting of pregnancy: prevalence and association with anti-emetic therapy. J Psychosom Obstet Gynaecol.

[8] O'Brien B, Evans M, White-McDonald E (2002). Isolation from "being alive": coping with severe nausea and vomiting of pregnancy. Nurs Res.

[9] Chou FH, Lin LL, Cooney AT, Walker LO, Riggs MW (2003). Psychosocial factors related to nausea, vomiting, and fatigue in early pregnancy. J Nurs Scholarsh.

[10] Dorheim S, Bjorvatn B, Eberhard-Gran M (2013). Sick leave during pregnancy: a longitudinal study of rates and risk factors in a Norwegian population. BJOG.

[11] Piwko C, Ungar WJ, Einarson TR, Wolpin J, Koren G (2007). The weekly cost of nausea and vomiting of pregnancy for women calling the Toronto Motherisk Program. Curr Med Res Opin.

[12] Piwko C, Koren G, Babashov V, Vicente C, Einarson TR (2013). Economic burden of nausea and vomiting of pregnancy in the USA. J Popul Ther Clin Pharmacol..

[13] Nordeng H, Ystrom E, Einarson A (2010). Perception of risk regarding the use of medications and other exposures during pregnancy. Eur J Clin Pharmacol.

[14] Einarson A, Koren G, Bergman U (1998). Nausea and vomiting in pregnancy: a comparative European study. Eur J Obstet Gynecol Reprod Biol.

[15] Lacasse A, Rey E, Ferreira E, Morin C, Berard A (2008). Nausea and vomiting of pregnancy: what about quality of life?. BJOG.

[16] Heitmann K, Nordeng H, Holst L (2013). Safety of ginger use in pregnancy: results from a large population-based cohort study. Eur J Clin Pharmacol.

[17] Asker C, Norstedt Wikner B, Kallen B (2005). Use of antiemetic drugs during pregnancy in Sweden. Eur J Clin Pharmacol.

[18] Hollyer T, Boon H, Georgousis A, Smith M, Einarson A (2002). The use of CAM by women suffering from nausea and vomiting during pregnancy. BMC Complement Altern Med.

[19] Chandra K, Magee L, Einarson A, Koren G (2003). Nausea and vomiting in pregnancy: results of a survey that identified interventions used by women to alleviate their symptoms. J Psychosom Obstet Gynaecol.

[20] Anderka M, Mitchell AA, Louik C, Werler MM, Hernandez-Diaz S, Rasmussen SA (2012). Medications used to treat nausea and vomiting of pregnancy and the risk of selected birth defects. Birth Defects Res A Clin Mol Teratol.

[21] van Gelder M, Pijpe A (2013). E-epidemiology: a comprehensive update. Cancer.

[22] Huybrechts KF, Mikkelsen EM, Christensen T, Riis AH, Hatch EE, Wise LA (2010). A successful implementation of e-epidemiology: the Danish pregnancy planning study ‘Snart-Gravid’. Eur J Epidemiol.

[23] Lupattelli A, Spigset O, Twigg M, Zagorodnikova K, Mårdby A, Moretti M (2014). Medication use in pregnancy: a cross-sectional, multinational web-based study. BMJ open.

[24] Martindale W, Sweetman SC (2011). Martindale: the complete drug reference.

[25] WHO Collaborating Centre for Drugs Statistics Methodology. Guidelines for ATC classification and DDD assignment 2013. http://www.whocc.no/atc_ddd_index/. Accessed 19 March 2015.

[26] Kennedy DA, Lupattelli A, Koren G, Nordeng H (2013). Herbal medicine use in pregnancy: results of a multinational study. BMC Complement Altern Med.

[27] Cox JL, Holden JM, Sagovsky R (1987). Detection of postnatal depression. Development of the 10-item Edinburgh Postnatal Depression Scale. Br J Psychiatry.

[28] Bergink V, Kooistra L, den Berg MP L-v, Wijnen H, Bunevicius R, van Baar A (2011). Validation of the Edinburgh Depression Scale during pregnancy. J Psychosom Res.

[29] Cox JL, Holden J. Perinatal mental health: a guide to the Edinburgh Postnatal Depression Scale (EPDS)*.* RCPsych Publications;2003.

[30] Department of Health (2006). Edinburgh Postnatal Depression Scale (EPDS): Translated versions – validated.

[31] Grote V, Vik T, von Kries R, Luque V, Socha J, Verduci E (2010). Maternal postnatal depression and child growth: a European cohort study. BMC pediatrics.

[32] Stewart DE, Gagnon A, Saucier JF, Wahoush O, Dougherty G (2008). Postpartum depression symptoms in newcomers. Can J Psychiatry.

[33] Tamminen T (1990). Postnatal depression, breastfeeding and mother-infant interaction. Unpublished doctoral thesis.

[34] Gadsby R, Barnie-Adshead T, Sykes C (2011). Why won't doctors prescribe antiemetics in pregnancy?. BMJ.

[35] Mazzotta P, Magee LA, Maltepe C, Lifshitz A, Navioz Y, Koren G (1999). The perception of teratogenic risk by women with nausea and vomiting of pregnancy. Reprod Toxicol.

[36] Maltepe C, Koren G (2013). The management of nausea and vomiting of pregnancy and hyperemesis gravidarum--a 2013 update. J Popul Ther Clin Pharmacol..

[37] Arsenault MY, Lane CA, MacKinnon CJ, Bartellas E, Cargill YM, Klein MC (2002). The management of nausea and vomiting of pregnancy. J Obstet Gynaecol Can.

[38] De Vigan C, De Walle HEK, Cordier S, Goujard J, Knill-Jones R, Ayme S (1999). Therapeutic drug use during pregnancy: A comparison in four European countries. J Clin Epidemiol.

[39] Goodwin TM, Poursharif B, Korst LM, MacGibbon KW, Romero R, Fejzo MS (2008). Secular trends in the treatment of hyperemesis gravidarum. Am J Perinatol.

[40] Seto A, Einarson T, Koren G (1997). Pregnancy outcome following first trimester exposure to antihistamines: meta-analysis. Am J Perinatol.

[41] Kallen B (2002). Use of antihistamine drugs in early pregnancy and delivery outcome. J Matern Fetal Neona.

[42] Pasternak B, Svanstrom H, Molgaard-Nielsen D, Melbye M, Hviid A (2013). Metoclopramide in pregnancy and risk of major congenital malformations and fetal death. JAMA.

[43] Matok I, Gorodischer R, Koren G, Sheiner E, Wiznitzer A, Levy A (2009). The safety of metoclopramide use in the first trimester of pregnancy. New Engl J Med.

[44] Smith J, Refuerzo J, Ramin S. Treatment and outcome of nausea and vomiting of pregnancy. UpToDate. 2014.

[45] National Collaborating Centre for Women's and Children's Health. Antenatal care: routine care for the healthy pregnant woman. http://www.nice.org.uk/guidance/cg62/resources/guidance-antenatal-care-pdf. Accessed 19 March 2015. 21370514

[46] ACOG (American College of Obstetrics and Gynecology) Practice Bulletin No. 153: Nausea and vomiting of pregnancy. Obstet Gynecol. 2015; 126(3):e12-24. 10.1097/AOG.0000000000001048 26287788

[47] Koren G. Scary Science: Ondansetron Safety in Pregnancy-Two Opposing Results From the Same Danish Registry. Therapeutic drug monitoring 2014. [Epub ahead of print]. 10.1097/FTD.0000000000000020 24413625

[48] European Medicines Agency. European Medicines Agency recommends changes to the use of metoclopramide. http://www.ema.europa.eu/docs/en_GB/document_library/Press_release/2013/07/WC500146500.pdf. Accessed 19 March 2015.

[49] FDA. Drug safety communication: Abnormal heart rythms may be associated with use of Zofran (ondansetron). http://www.fda.gov/Drugs/DrugSafety/ucm271913.htm. Accessed 19 March 2015.

[50] Pasternak B, Svanstrom H, Hviid A (2013). Ondansetron in pregnancy and risk of adverse fetal outcomes. The New England journal of medicine.

[51] Andersen JT, Jimenez-Solem E, Andersen NL, Poulsen HE (2013). Ondansetron use in early pregnancy and the risk of congenital malformations-a register based nationwide cohort study. Pharmacoepidemiol Drug Saf.

[52] Forster DA, Denning A, Wills G, Bolger M, McCarthy E (2006). Herbal medicine use during pregnancy in a group of Australian women. BMC Pregnancy Childbirth.

[53] Frawley J, Adams J, Sibbritt D, Steel A, Broom A, Gallois C (2013). Prevalence and determinants of complementary and alternative medicine use during pregnancy: results from a nationally representative sample of Australian pregnant women. Aust N Z J Obstet Gynaecol.

[54] Dante G, Pedrielli G, Annessi E, Facchinetti F (2013). Herb remedies during pregnancy: a systematic review of controlled clinical trials. J Matern Fetal Neonatal Med.

[55] Kraft K (1997). Artichoke leaf extract - Recent findings reflecting effects on lipid metabolism, liver and gastrointestinal tracts. Phytomedicine.

[56] Louik C, Hernandez-Diaz S, Werler MM, Mitchell AA (2006). Nausea and vomiting in pregnancy: maternal characteristics and risk factors. Paediatr Perinat Epidemiol.

[57] Kallen B, Lundberg G, Aberg A (2003). Relationship between vitamin use, smoking, and nausea and vomiting of pregnancy. Acta obstetricia et gynecologica Scandinavica.

[58] Weigel MM, Weigel RM (1988). The association of reproductive history, demographic factors, and alcohol and tobacco consumption with the risk of developing nausea and vomiting in early pregnancy. Am J Epidemiol.

[59] Emelianova S, Mazzotta P, Einarson A, Koren G (1999). Prevalence and severity of nausea and vomiting of pregnancy and effect of vitamin supplementation. Clinical and investigative medicine Medecine clinique et experimentale.

[60] Kramer J, Bowen A, Stewart N, Muhajarine N (2013). Nausea and vomiting of pregnancy: prevalence, severity and relation to psychosocial health. MCN Am J Matern Child Nurs.

[61] Gill SK, Maltepe C, Koren G (2009). The effect of heartburn and acid reflux on the severity of nausea and vomiting of pregnancy. Can J Gastroenterol.

[62] Gill SK, Maltepe C, Mastali K, Koren G (2009). The effect of Acid-reducing pharmacotherapy on the severity of nausea and vomiting of pregnancy. Obstet Gynecol Int.

[63] Ekman A, Dickman PW, Klint A, Weiderpass E, Litton JE (2006). Feasibility of using web-based questionnaires in large population-based epidemiological studies. Eur J Epidemiol.

[64] van Gelder MM, Bretveld RW, Roeleveld N (2010). Web-based questionnaires: the future in epidemiology?. Am J Epidemiol.

[65] Seybert H. Internet use in households and by individuals in 2011. http://ec.europa.eu/eurostat/documents/3433488/5579964/KS-SF-11-066-EN.PDF/090e071f-c3a9-45d8-aa90-9b142251fd3a?version=1.0. Accessed 19 March 2015.

[66] Internet World Stats. Usage and population statistics. 2012. Available from: http://www.internetworldstats.com/. Accessed 19 March 2015.

[67] United States Census Bureau. The 2012 Statistical Abstract. Information & Communications; 2010. Available from: http://www.census.gov/compendia/statab/cats/information_communications/internet_publishipu_and_broadcasting_and_internet_usage.html. Accessed 19 March 2015.

