# Supplementary material for: Treatment of nausea in pregnancy: a cross-sectional multinational web-based study of pregnant women and new mothers
Source: BMC Pregnancy Childbirth. 2015 Dec 1;15:321. doi: 10.1186/s12884-015-0746-2 (PMC4667480; doi:10.1186/s12884-015-0746-2)
Supplement: Additional file 1: — STROBE Statement—Checklist of items that should be included in reports of cross-sectional studies. (PDF 96 kb) [file 12884_2015_746_MOESM1_ESM.pdf]

Additional file 1: Most common medicines used against nausea on 1st and 2nd ATC level according to timing of use in pregnancy among women with nausea in pregnancy (n=6701)*

Anatomical Therapeutic Chemical (ATC) classification index 1st and 2nd levels		Anytime during pregnancy n (%)	1st trimester n (%)	2nd trimester n (%)	3rd trimester n (%)
**A**	**Alimentary tract and metabolism**	**564 (8.4)**	**448 (6.7)**	**249 (3.7)**	**141 (2.1)**
**A02**	Drugs for acid related disorders	176 (2.6)	105 (1.6)	83 (1.2)	78 (1.2)
**A03**	*Drugs for functional gastrointestinal disorders*	316 (4.7)	277 (4.1)	137 (2.0)	52 (0.8)
**A04**	Antiemetics and antinauseants	90 (1.3)	81 (1.2)	47 (0.7)	22 (0.3)
**A05**	Bile and liver therapy	12 (0.2)	9 (0.1)	6 (0.1)	1 (0.0)
**A07**	Antidiarrheals, intestinal antiinflammatory/antiinfective agents	17 (0.3)	13 (0.2)	5 (0.1)	2 (0.0)
**N**	**Nervous system**	**115 (1.7)**	**81 (1.2)**	**74 (1.1)**	**34 (0.5)**
**N02**	*Analgesics*	83 (1.2)	54 (0.8)	52 (0.8)	24 (0.4)
**N05**	Psycholeptics	32 (0.5)	27 (0.4)	22 (0.3)	10 (0.2)
**R**	**Respiratory system**	**620 (9.3)**	**536 (8.0)**	**319 (4.8)**	**111 (1.7)**
**R06**	*Antihistamines for systemic use*	613 (9.2)	533 (8.0)	316 (4.7)	108 (1.6)
**-**	**Unspecified**	82 (1.2)	57 (0.9)	46 (0.7)	17 (0.3)
**Total medicine use (any ATC)**		**1201 (17.9)[a]**	**965 (14.4)**	**577 (8.6)**	**254 (3.8)**
**Total vitamin and/or mineral use** *(ATC-class: A11-13, B03 and Unspecified supplement)*		**106 (1.6)**	**90 (1.3)**	**42 (0.6)**	**9 (0.1)**

*Only medicine groups used by more than 10 women are presented. Exposure timing is defined as follows: 1st trimester (gestational weeks 0-12), 2nd trimester (gestational week 13-24), 3rd trimester (gestational week 25 and up to childbirth).

[a] Numbers do not add up to total due to some women may have used more than one medicine
